# Supplementary material for: Depletion of extracellular asparagine impairs self-reactive T cells and ameliorates autoimmunity in a murine model of multiple sclerosis
Source: bioRxiv. 2025 Jun 11:2025.06.09.658561. Preprint. [Version 1] doi: 10.1101/2025.06.09.658561 (PMC12259033; doi:10.1101/2025.06.09.658561)
Supplement: 1 [file NIHPP2025.06.09.658561V1-supplement-1.pdf]

bioRxiv preprint doi: https://doi.org/10.1101/2025.06.09.658561; this version posted June 11, 2025. The copyright holder for this preprint (which was not certified by peer review) is the author/funder, who has granted bioRxiv a license to display the preprint in perpetuity. It is made available under aCC-BY 4.0 International license.

S1

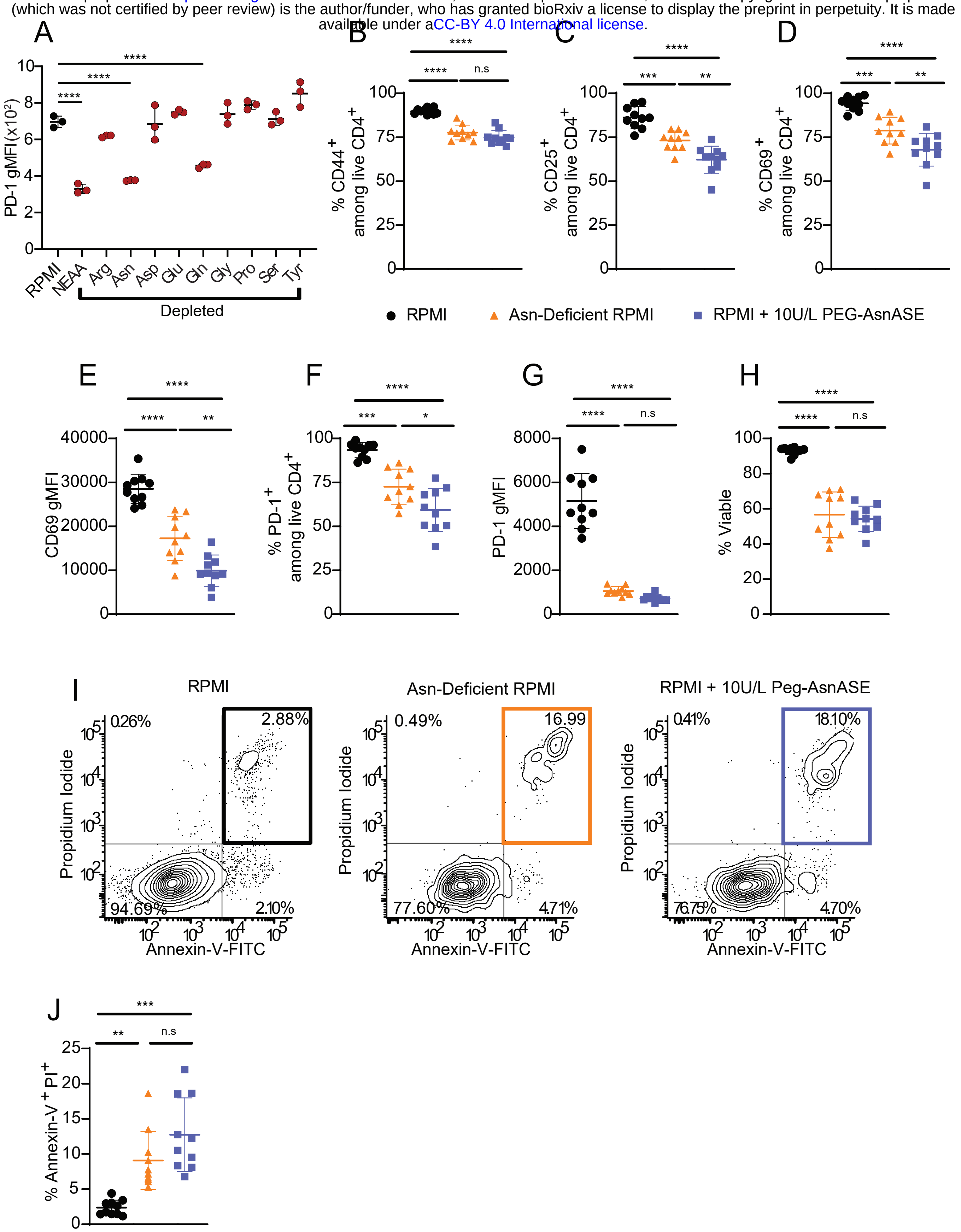

bioRxiv preprint doi: https://doi.org/10.1101/2025.06.09.658561; this version posted June 11, 2025. The copyright holder for this preprint (which was not certified by peer review) is the author/funder, who has granted bioRxiv a license to display the preprint in perpetuity. It is made available under aCC-BY 4.0 International license.

Supplemental Figure 1**. Extracellular asparagine is essential for CD4$^+$ T cell activation.**

(**A**) Expression levels of the activation marker PD-1 following 24 hours of stimulation with plate-bound anti-CD3/CD28 mAbs in RPMI lacking the indicated individual amino acids shown in red. Non-essential amino acids (NEAA) include asparagine (Asn), aspartate (Asp), glutamate (Glu), proline (Pro), arginine (Arg), glutamine (Gln), glycine (Gly), serine (Ser), and tyrosine (Tyr). Quantification of the proportions of CD4$^+$ T cells expressing cell surface activation markers CD44 (**B**), CD25 (**C**), CD69 (**D**), and PD-1 (**F**) as well as expression levels of CD69 (**E**) and PD-1 (**G**) on a per cell basis following 24 hours of stimulation with plate-bound anti-CD3/CD28 mAbs in complete RPMI (RPMI), Asn-deficient RPMI or RPMI with 10 IUs/L PEGylated-asparaginase (PEG-AsnASE) added at the start of culture. (**H**) Quantification of the proportions of viable CD4$^+$ T cells in each respective culture condition after 3 days of stimulation with plate-bound anti-CD3/CD28 mAbs in RPMI, Asn-deficient RPMI or RPMI with 10 IUs/L PEG-AsnASE added at the start of culture. (**I**) Representative flow cytometry contour plots depicting Annexin-V and propidium iodide staining in naive CD4$^+$ T cell following 2 days of stimulation with plate-bound anti-CD3/CD28 mAbs in RPMI, Asn-deficient RPMI or RPMI with 10 IUs/L PEG-AsnASE added from the initiation of culture. Upper right quadrants depict the proportions of CD4$^+$ T cells that have undergone apoptosis in each respective culture condition. (**J**) Quantification of the proportion of Annexin-V propidium iodide double-positive CD4$^+$ T cells as shown in I. Each dot represents cells obtained from an individual animal (B-H, J). Results are shown as mean ± SD and are pooled from 2 independent experiments (B-H, J) or a representative of 2 independent experiments (A, I). non-significant (n.s), *p < 0.05 **p < 0.01, ***p < 0.001, ****p < 0.0001, one-way ANOVA with Turkey's multiple comparison test (B-H, J) or Dunnet's multiple comparison test (A).

S2 bioRxiv preprint doi: https://doi.org/10.1101/2025.06.09.658561; this version posted June 11, 2025. The copyright holder for this preprint (which was not certified by peer review) is the author/funder, who has granted bioRxiv a license to display the preprint in perpetuity. It is made available under aCC-BY 4.0 International license.

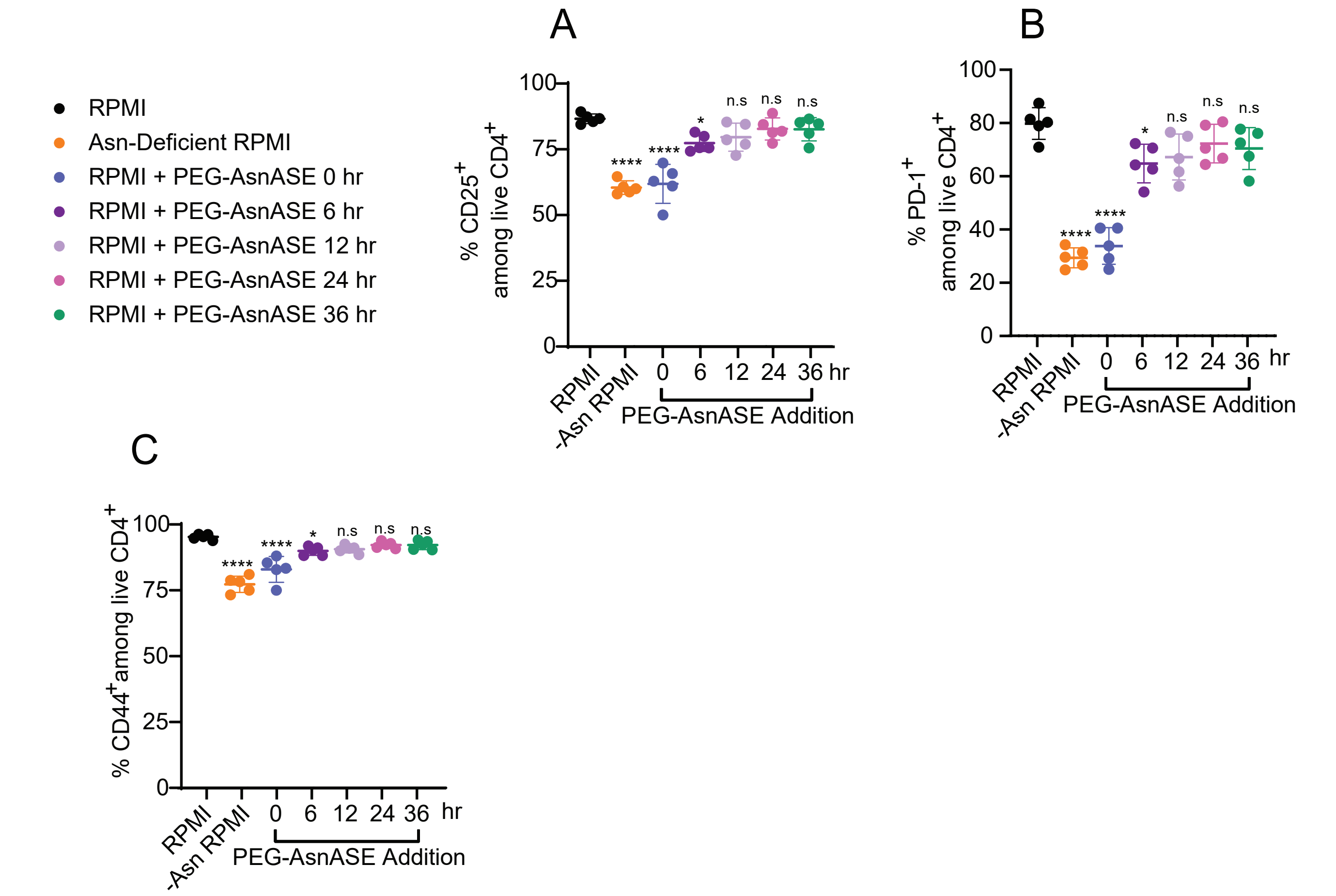

bioRxiv preprint doi: https://doi.org/10.1101/2025.06.09.658561; this version posted June 11, 2025. The copyright holder for this preprint (which was not certified by peer review) is the author/funder, who has granted bioRxiv a license to display the preprint in perpetuity. It is made available under aCC-BY 4.0 International license.

Supplemental Figure 2. **Expression of CD4$^+$ T cell activation proteins in asparagine sufficient and deficient conditions.**

(**A-C**) Quantification of the proportions of CD4$^+$ T cells expressing CD25 (**A**), PD-1 (**B**), and CD25 (**C**) following 2 days of stimulation with plate-bound anti-CD3/CD28 mAbs in complete RPMI, Asn-deficient RPMI or RPMI with 10 IUs/L PEGylated-asparaginase added at 0, 6, 12, 24, or 36 hours. Each dot represents cells obtained from an individual animal (A-C). Results are shown as mean ± SD and are representative of 2 independent experiments non-significant (n.s), *$p < 0.05$, ****$p < 0.0001$, one-way ANOVA with Dunnet's multiple comparison test.

S3

bioRxiv preprint doi: https://doi.org/10.1101/2025.06.09.658561; this version posted June 11, 2025. The copyright holder for this preprint (which was not certified by peer review) is the author/funder, who has granted bioRxiv a license to display the preprint in perpetuity. It is made available under aCC-BY 4.0 International license.

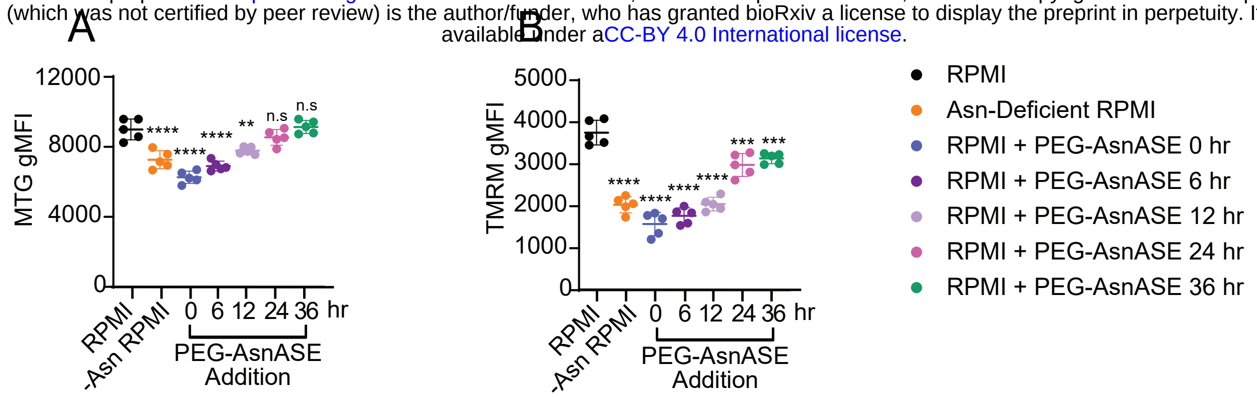

bioRxiv preprint doi: https://doi.org/10.1101/2025.06.09.658561; this version posted June 11, 2025. The copyright holder for this preprint (which was not certified by peer review) is the author/funder, who has granted bioRxiv a license to display the preprint in perpetuity. It is made available under aCC-BY 4.0 International license.

Supplemental Figure 3. **Asparagine deprivation impairs mitochondrial function in CD4$^+$ T cells.**

(**A-B**) Naive CD4$^+$ T cells were stimulated for 48 hours with plate-bound anti-CD3/CD28 mAbs in either complete RPMI media (RPMI), Asn-deficient RPMI or RPMI treated with 10 IUs/L PEGylated-asparaginase (PEG-AsnASE) added at 0, 6, 12, 24, or 36 hours. (**A**) Quantification of mitotracker green (MTG) gMFI. (**B**) Quantification of tetramethyl rhodamine methyl ester (TMRM) gMFI. Results are shown as mean ± SD and are representative of at least 2 independent experiments. non-significant (n.s), **p < 0.01, ***p < 0.001, ****p < 0.0001, one-way ANOVA with Dunnet's multiple comparison test.

S4

bioRxiv preprint doi: https://doi.org/10.1101/2025.06.09.658561; this version posted June 11, 2025. The copyright holder for this preprint (which was not certified by peer review) is the author/funder, who has granted bioRxiv a license to display the preprint in perpetuity. It is made available under aCC-BY 4.0 International license.

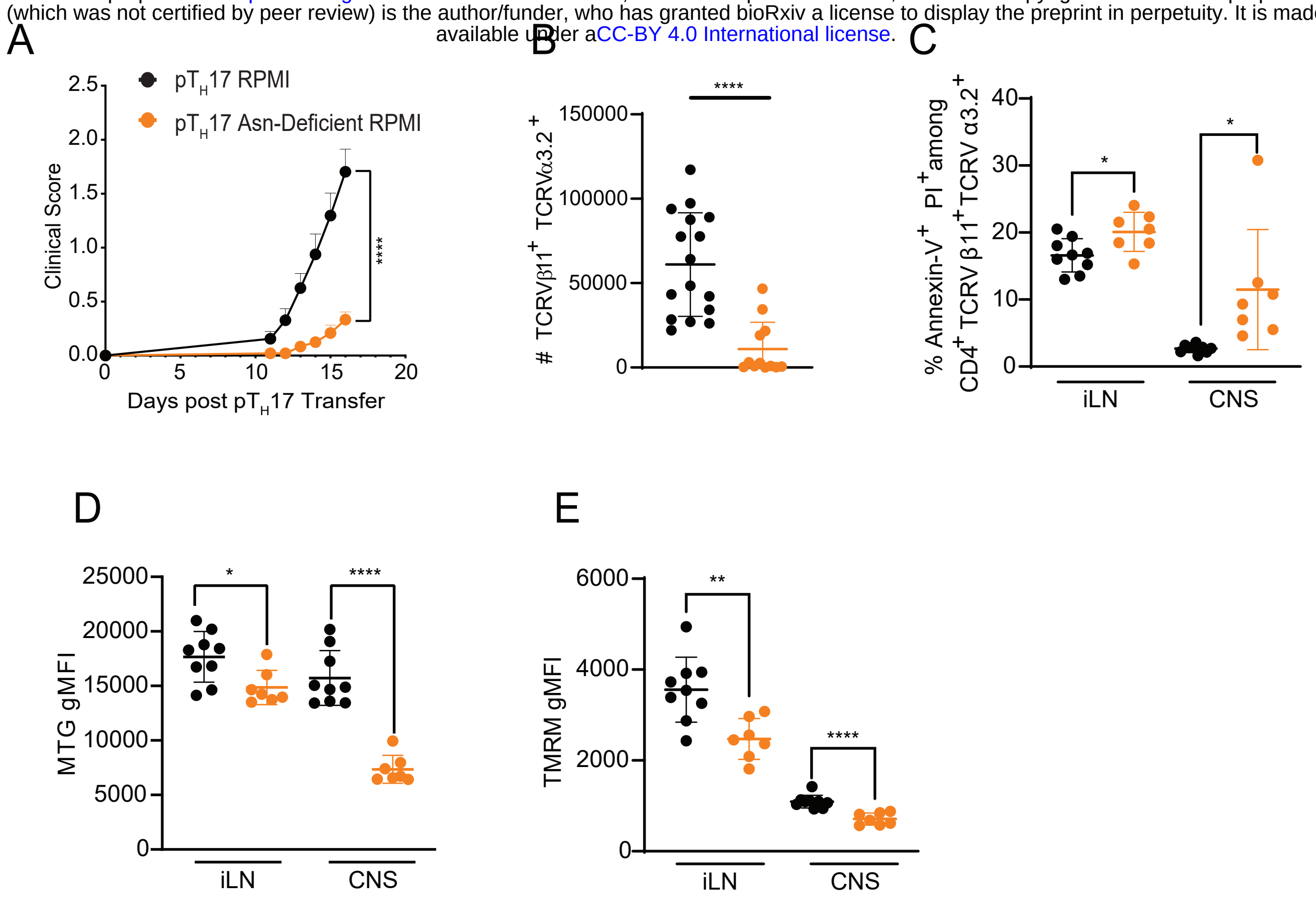

bioRxiv preprint doi: https://doi.org/10.1101/2025.06.09.658561; this version posted June 11, 2025. The copyright holder for this preprint (which was not certified by peer review) is the author/funder, who has granted bioRxiv a license to display the preprint in perpetuity. It is made available under aCC-BY 4.0 International license.

Supplemental Figure 4. **Asparagine deprivation impairs pT_H17 function in a model of induced EAE.**

(**A)** Pathogenic T helper 17 (pT_H17) cells were differentiated from naive $CD4^+$ $FoxP3^-$ T cells from 2D2 TCR transgenic mice in RPMI media with or without Asn. Viable pT_H17 polarized 2D2 cells were adoptively transferred ($4x10^6$/mouse) into 10-week-old C57BL/6J female recipients to induce EAE. Mice were scored daily for signs of disease. (**B**) At peak EAE (Day 16), 2D2 cells were isolated from the CNS and inguinal lymph (iLN) node and analyzed. Quantification of the absolute numbers of central nervous system (CNS)-infiltrating $V\beta11^+V\alpha3.2^+$ 2D2 pT_H17 cells at peak of EAE (pT_H17 RPMI n=16, pT_H17 Asn-deficient RPMI n=12). (**C**) Quantification of the proportions of $V\beta11^+V\alpha3.2^+$ 2D2 pT_H17 cells that have undergone apoptosis (Annexin-V^+PI^+) in the CNS and iLN node at peak of EAE. (**D-E**) Mitotracker green (MTG) and tetramethyl rhodamine methyl ester (TMRM) gMFI in iLN and CNS infiltrating $TCRV\beta11^+TCRV\alpha3.2^+$ $CD4^+$ T cells at peak of EAE. Each dot represents an individual mouse (B-E). Results are shown as mean ± SD and are pooled (A-B) or a representative of at least 2 independent experiments (C-E). non-significant (n.s), *$p < 0.05$ **$p < 0.01$, ****$p < 0.0001$, two-way ANOVA (A), and Student's t-test (B-E).
